# Pneumonia: Does Age or Gender Relate to the Presence of an SLP Dysphagia Consultation?

**DOI:** 10.3390/geriatrics5030051

**Published:** 2020-09-14

**Authors:** Christopher Bolinger, James Dembowski, Kimberly Mory

**Affiliations:** 1 Department of Communication Sciences and Oral Health, College of Health Sciences, Texas Woman’s University, Denton, TX 76207, USA; kmory@twu.edu; 2 Department of Speech, Language, and Hearing Sciences, School of Health Professions, Texas Tech Health Sciences Center, Lubbock, TX 79430, USA; james.dembowski@ttuhsc.edu

**Keywords:** dysphagia, geriatrics, pneumonia

## Abstract

A retrospective study of 1489 hospital records examined the relationship of speech-language pathologist (SLP) consults for dysphagia to age and gender in pneumonia patients from an acute care setting. Age and gender disparities exist in healthcare. The research sought to determine if disparities existed in the presence/absence of SLP dysphagia consults related to age and gender. Results suggested SLPs were consulted on a greater percentage of geriatric patients overall; however, there were differences in the number of consults for each pneumonia type. More males and geriatric patients were seen than females and non-geriatric adults, respectively. Results may be used to address local hospital policies and protocols and thus increase quality of care by improving morbidity and mortality outcomes of geriatric patients with pneumonia.

## 1. Introduction

Age and gender disparities are known to exist in the current healthcare system of the United States [1,2]. This is increasingly alarming with the current population statistics in the country. According to Roberts et al. [3], there were 617.1 million geriatric people living in the world. The researchers projected this to increase to approximately one billion worldwide. According to the US Census Bureau’s 2016 American Community Survey, there are 49.2 million Americans 65 years of age or older [3]. According to the survey, there were 79 males to every 100 females in the geriatric population. As an advanced society, this growing population’s healthcare disparities must be addressed.

The researchers sought to determine if a disparity existed in the consultation of speech-language pathologists for possible dysphagia in older individuals admitted to an acute-care hospital with a pneumonia diagnosis. Dysphagia refers to a broad classification of swallowing disorders [4]. As an individual ages, the efficacy of the swallow function diminishes [5]. Prior research of individuals with respiratory conditions identified an increased risk of aspiration secondary to the coordination of the swallowing and breathing [6]. In the acute-care hospital setting, dysphagia is commonly associated with the following co-morbidities: dehydration, esophageal disease, stroke (cerebral vascular accident), aspiration pneumonia, urinary tract infection, and congestive heart failure [7]. According to Hayashi et al. [8], pneumonia is the leading cause of death in the geriatric population. Since dysphagia is associated with pneumonia, evaluation of swallow function should be included as part of the differential diagnosis and not dismissed as part of the aging swallow. Therefore, the researchers sought to identify a specific area to improve the current standards of care in treating the geriatric population with pneumonia.

For geriatric patients with community-acquired pneumonia and health care-acquired pneumonia, dysphagia increases the risk of aspiration pneumonia [9,10]. The risks increase with age and comorbidities. According to Teramoto et al. [10], aspiration pneumonia is responsible for up to 66.8% of pneumonia hospitalizations. Out of 589 pneumonia cases reviewed, over 81% were in geriatric patients [10]. Other studies have found that over 75% of geriatric patients with community-acquired pneumonia have dysphagia [11].

The long-range goals of this project were to improve the quality of health care for individuals diagnosed with pneumonia and identify any disparities as they relate to gender and age. Specific aim 1 sought to identify any differences in age for patients with pneumonia when speech-language pathology was, and was not, consulted. Specific aim 2 sought to identify any differences in gender for patients with pneumonia when speech-language pathology was, and was not, consulted.

Based upon previous research [9,10], the researchers hypothesized more consults for geriatric populations admitted with aspiration pneumonia and more consults for males admitted with aspiration pneumonia. However, the researchers also hypothesized that geriatric populations with non-aspiration pneumonia would receive disproportionally less consults as their dysphagia may be dismissed as an aging swallow.

## 2. Materials and Methods

### 2.1. Research Design

This study was a retrospective exploratory and descriptive investigation of a sample of persons with a diagnosis of pneumonia admitted to a west Texas university-affiliated medical center. The Texas Tech University Health Sciences Center Institutional Review Board approved the study (IRB# L15-056). A waiver of informed consent was approved in accordance with 45CFR 46.116(d) and 46.164.512(i)(1)(iii). The dependent variables for this study were patient pneumonia diagnosis type and speech pathology consultation. The independent variables were the demographic characteristics of age and gender. The study sought to determine if these demographic characteristics influenced the likelihood of an SLP consultation for dysphagia concerns.

Pneumonia diagnosis was a categorical variable coded as one of four types indicated in the medical chart: organism unspecified by the physician, health care-acquired, aspiration, and community-acquired. Speech-language pathology consultation was a categorical variable: consultation placed, or no consultation placed. Age was operationalized through a categorical binary code: adults 18 years of age to 60 years of age or geriatrics 61 years of age and older. Categories for age were based upon Gorman’s [12] research in patient rights of older persons. Gender was a categorical variable: male or female.

### 2.2. Data Collection

Medical charts for review were identified by requesting a report from the hospital’s Information Technology/Medical Records department, through the institution’s Clinical Research Institute (CRI). Admission date range, age, and pneumonia diagnosis were the criteria that formed the basis of the chart identifications. Patients admitted to the hospital between 1 November 2013, and 1 November 2014, were included in the study. The date range (November to November for a single year) was used to incorporate all seasons of the year and eliminate confounding variables such as influenza season. Of the patients included, all were males and females between the ages of 18–99 years. Each patient had one of the following four diagnosis on admission to the hospital:

1. Pneumonia—organism unspecified (ICD 9-486);
2. Health Care Acquired pneumonia—pneumonia due to staphylococcus (ICD 9-482.4x) or ventilator-associated (ICD 9-997.31);
3. Aspiration pneumonia—pneumonia due to aspirated foods, liquids, medications (ICD 9-507.x);
4. Community acquired pneumonia—bacteria pneumonia unspecified (ICD 9-482.9).

### 2.3. Participants

The study included 1489 individuals diagnosed with pneumonia. Consent for this retrospective study of medical records was not obtained because it would create an additional and unnecessary link between participants and the study. All data were de-identified following extraction from the medical charts. Participants for the study ranged in age from 18 to 99 years. The mean age of participants in the study was 51.5 years (SD = 4.95 years). Six hundred eighty patients fell into the non-geriatric adult category, and 809 patients were categorized as geriatric. Males numbered 798, and females numbered 691. Race was not considered secondary to the questionable accuracy of the categorization of race in patient reports. Education and socioeconomic status were not noted in the medical records, so these variables were not included in this study.

### 2.4. Statistical Analysis

Descriptive statistics are reported in terms of the type of pneumonia diagnosis. Correlations explored the relationship between incidence values and the presence or absence of speech-language pathology consultations. The phi coefficient was used to examine associations between two dichotomous variables. Measures that suggested distinctive differences were subjected to *t*-tests. Pearson’s chi-squared analysis, using a one-tailed definition, was utilized to determine if differences between data categories could be explained through chance. A binomial logistic regression was completed to further analyze the relationships. Statistical analysis was performed using the Statistical Package for the Social Sciences (SPSS^®^) Version 22 [13].

## 3. Results

Age. Table 1 shows numbers and proportions of age groups of patients with pneumonia in both categories: without SLP consult and with SLP consult. A larger proportion of geriatric patients received an SLP consult (58.55% versus 41.45%). As both variables are dichotomous, the phi coefficient was used. The phi coefficient is another special case of the Pearson product-moment correlation. A test of the phi coefficient showed that speech-language pathology consultation is related to age category for all combined pneumonia cases. Specifically, patients in the geriatric category are more likely to get an SLP consult for dysphagia. The likelihood that this does not occur by chance is significant (r**_ϕ_** = 0.0573, *p* = 0.0271). The odds of a geriatric pneumonia patient having a speech-language pathology consult for dysphagia are 1.117 times greater than an adult pneumonia patient. A chi-square test was performed and confirmed a relationship between speech-language pathology consults and age group, X2 (2, *n* = 1489) = 4.888, *p* = 0.027. Even though there seems to be a relatively small difference (6%) between the geriatric patients who received an SLP consult and those who did not, within the category of patient who received an SLP consult, geriatrics received a larger proportion of consultations (17.1% greater). That is, 58.5% of the consultations were geriatric patients versus 41.45% of adult patients between 18 and 60 years of age.

When the pneumonia categories are analyzed further (see Table 2), there is a statistical significance for the presence/absence of SLP dysphagia consults in community-acquired pneumonia as related to the age categories (*p* = 0.003). Geriatric patients admitted to the hospital with community-acquired pneumonia were more likely to receive an SLP dysphagia consult. The likelihood that this does not occur by chance is significant (r**_ϕ_** = 0.190, *p* = 0.003). However, there is no statistical significance (*p* > 0.05) in SLP dysphagia consults for the other categories of pneumonia (i.e., organism not specified, health care-acquired, and aspiration) related to the age categories. There was a very weak correlation for an SLP consult for age and organism not specified pneumonia, though not statistically significant (r**_ϕ_** = 0.025, *p* = 0.455). There was no correlation or statistical significance for an SLP consult for age and health care-acquired pneumonia (r**_ϕ_** = −0.018, *p* = 0.853). There was a moderate correlation for an SLP consult for age and aspiration pneumonia, though not statistically significant (r**_ϕ_** = 0.129, *p* = 0.055).

Gender. Table 3 shows number and proportions of gender groups of patients with pneumonia in both categories: without SLP consult and with SLP consult. A larger proportion of male patients received an SLP consult as opposed to female patients with pneumonia (58.76% versus 41.24%). A test of the phi coefficient showed an association between speech-language pathology consultation and gender. The likelihood that this association does not occur by chance is significant (r**_ϕ_** = −0.0702, *p* = 0.0068). The odds of a male pneumonia patient having a speech-language pathology consult are 1.147 times greater than a female pneumonia patient. A chi-square test was performed and confirmed a relationship between speech-language pathology consults and gender groups, X2 (2, *n* = 1489) = 7.328, *p* = 0.007. Even though there seems to be a small difference (7.54%) between the male patients who received an SLP consult and those who did not, within the category of patients who received an SLP consult, males received a larger proportion of consultations (17.52% greater). That is 58.76% were males versus 41.24% of females. This is a difference of 17.52%.

**Figure 1:**
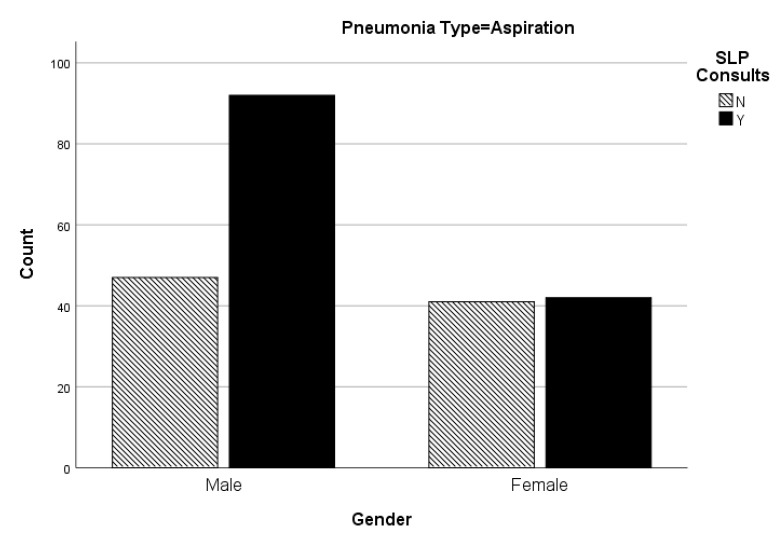
Number of SLP consults from each age category for aspiration pneumonia.

When the pneumonia categories are analyzed further (see Table 4), there is a statistical significance for the presence/absence of SLP dysphagia consults in aspiration pneumonia as related to the gender categories (*p* = 0.022). Male patients admitted to the hospital with aspiration pneumonia were more likely to receive an SLP dysphagia consult (see Figure 1). The likelihood that this does not occur by chance is significant (r**_ϕ_** = 0.154, *p* = 0.022). However, there is no statistical significance (*p* > 0.05) in SLP dysphagia consults for the other categories of pneumonia (i.e., organism not specified, health care-acquired, and community-acquired) related to the gender categories. There was a very weak correlation for an SLP consult for gender and organism not specified pneumonia, though not statistically significant (r**_ϕ_** = 0.013, *p* = 0.694). There was a moderate correlation but no statistical significance for an SLP consult for gender and health care-acquired pneumonia (r**_ϕ_** = −0.11, *p* = 0.25). There was a weak correlation for an SLP consult for gender and community-acquired pneumonia, though not statistically significant (r**_ϕ_** = 0.06, *p* = 0.344).

Binomial logistic regression was completed on the variables. A binomial logistic regression was calculated to predict SLP consult based on age and gender. Results indicated that once variables were controlled for, there was a relationship between consults, age, and gender (*p* < 0.05). Non-geriatric adults were 0.769 times less likely to receive an SLP consult, while males were 1.372 times more likely to receive a consult (see Table 5).

## 4. Discussion

Specific Aim #1 sought to identify any differences in age for patients with pneumonia when speech-language pathology was, and was not, consulted. The results suggested speech-language pathologists saw a larger number of geriatric pneumonia patients versus non-geriatric adult pneumonia patients. This seems intuitive in that it would be expected that older individuals have more health concerns. An impaired swallow in the geriatric population would be expected due to the fact that with normal aging, our muscles become weaker. As Daniels et al. [14] found, persons are more prone to upper-airway penetration with increased age. However, though more health concerns and increased muscle weakness are noted in the geriatric population, this does not negate the fact that any individual with an impaired swallow, despite etiology or age, is at an increased risk of aspiration pneumonia. Therefore, patients with compromised respiratory systems have a weakened immune system secondary to illness. These patients are more likely to develop aspiration pneumonia regardless of age category in which they are associated.

It is concerning that the only category that showed a correlation and statistical significance between SLP consult and age was community-acquired pneumonia. Both health care-acquired and aspiration pneumonia have specific etiologies, one may identify swallow dysfunctions based on differential diagnosis techniques; however, organism not specified pneumonia is a broad classification with a multitude of underlying etiologies. When a patient is admitted to the hospital with pneumonia, the physician may initially classify the pneumonia as “organism not specified” until a differential diagnosis is completed using a battery of examinations, observations, and test results. This general classification of pneumonia account for approximately two-thirds of the pneumonia cases in the study (913 of 1489). Therefore, based upon increased risk factors associated with the aging population, more SLP dysphagia consults would be expected to contribute to the differential diagnosis. The SLP could also teach the patient aspiration risk reduction techniques and provide therapeutic intervention to increase strength and efficacy of the aging swallow function. This is especially important since previous research has suggested that when a patient has other types of pneumonia, the risks of developing aspiration pneumonia and subsequent re-admissions to the hospital is a key concern [9,10]. SLPs are specially trained to identify and treat dysphagia. Since eating and drinking contribute to quality of life, it may be considered essential to assist the patient in a safe and effective manner. SLPs are able to provide compensatory strategies to aid in risk reduction of developing aspiration pneumonia. In some patients, SLPs may be able to provide a therapeutic plan that can increase speed and efficiency of the swallow function. However, if the physician is not consulting the SLP for a dysphagia evaluation, then the patient will not be receiving a complete plan of care. One way to address this concern is to develop a standardized order set. This means that when a geriatric patient is admitted with any type of pneumonia, the SLP is consulted to assess the safety and efficacy of the swallow function. Most healthcare providers acknowledge that early intervention is key to successfully treating most conditions. Therefore, it is not unreasonable to apply this school of thought to dysphagia.

Specific Aim #2 sought to identify any differences in gender for patients with pneumonia when speech-language pathology was, and was not, consulted. The results of the study indicated more speech-language pathology consults were placed for males with pneumonia as opposed to females with pneumonia. A new study published in July 2020 suggested significant differences in types of dysphagia between genders [15]. Males had more oropharyngeal dysphagia, while females had more esophageal dysphagia [15]. Males tend to seek medical attention for dysphagia at a higher proportion than females [16]. One may argue that this is a sociological factor based upon gender roles. Females, historically, are the nurturers and caregivers; therefore, their personal health is often neglected. When admitted to the hospital, they may opt to minimize symptoms to allow for a quicker discharge and resume daily responsibilities as caregivers [17]. It is also well documented that women report more chronic diseases such as arthritis, hypertension, diabetes, COPD, and visual impairment than men, and seek preventative care less frequently than men [17]. Researchers found that men tend to seek medical intervention for acute illnesses more often than women and may exhibit better recoveries secondary to less concomitant conditions [18]. Speech-language pathologists are accustomed to treating more males than females for certain disorders (e.g., stuttering, autism, and language delays); however, both genders are equally susceptible to pneumonia.

The results of the research identified more SLP dysphagia consultations with males diagnosed with aspiration pneumonia as compared to females in the same pneumonia category. This is concerning in that the etiologies of aspiration pneumonia are highly correlated to dysphagia. That is, swallow dysfunction is one of the most common causes of aspiration pneumonia. Therefore, there should have been no statistical difference between the two genders.

## 5. Implications and Future Research

This study suggests further education regarding gender and age and the implications of aspiration pneumonia should be provided to the medical community. Lanspa et al. [19] reported in a study of 5185 patients that those with aspiration pneumonia were more than twice as likely to die in an inpatient setting as those patients with non-aspiration pneumonia. Research is needed to examine the susceptibility of women with chronic medical conditions developing aspiration pneumonia compared to males who report less chronic conditions. As more healthcare providers are educated regarding risks and consequences of dysphagia, the disparities seen in gender and age should start to dissipate. This research adds to current knowledge base.

However, the limitations of the research should also be acknowledged. The diagnosis of pneumonia was not standardized, the presence/absence of dysphagia was not identified, and the therapeutic intervention from the SLP was not identified. Another limitation of this study was that it was conducted using only data from one facility. A multi-site project would allow for improved generalization. This research serves as pilot data for a perspective study. Future studies may use a standardized pneumonia diagnosis and severity tool (e.g., CURB). In addition, a dysphagia screening tool will be used on all patients admitted with pneumonia. Failure on the screening tool would initiate an SLP dysphagia consult. All patients would receive an instrumental swallow evaluation to reduce the subjectiveness of the SLP evaluation. SLP interventions would be categorized into groups (e.g., rehabilitative intervention and compensatory intervention), and duration of interventions would be documented. In addition, future research will track patient outcomes (i.e., length of stay, morbidity, mortality, and 30-day readmission rates). This will allow for a more detailed statistical analysis.

Based on the data from this study, further research is warranted to determine if age and gender disparities in patients with pneumonia exist nationwide or if this was isolated to the facility in which the records were analyzed. In addition, further research is warranted regarding the patient outcomes (e.g., length of stay, mortality, morbidity, and 30-day readmission rates) in the specific age and gender categories.

## Figures and Tables

**Table 1:** Percentage of speech-language pathologist (SLP) consults from each age category.

	Age		Total
	Adult	Geriatric	
	Number	Number	Number
**without SLP consult**	486 (47.60%)	535 (52.40%)	1021 (100%)
**with SLP consult**	194 (41.45%)	274 (58.55%)	468 (100%)

**Table 2:** Categories and age.

Pneumonia Type	Without SLP Consult	With SLP Consult	Phi Coefficient	
			r_ϕ_	Significance
**Organism Not Specified**	651	262	0.025	0.455
***Non-Geriatric Adults***	291	110	---	---
***Geriatric Adults***	360	152	---	---
**Health Care-Acquired**	77	32	0.018	0.853
***Non-Geriatric Adults***	37	16	---	---
***Geriatric Adults***	40	16	---	---
**Aspiration**	88	134	0.129	0.055
***Non-Geriatric Adults***	49	57	---	---
***Geriatric Adults***	39	77	---	---
**Community-Acquired**	205	40	0.190	0.003 ***
***Non-Geriatric Adults***	109	11	---	---
***Geriatric Adults***	96	29	---	---
**Total**	1021	468	0.057	0.027 ***
***Non-Geriatric Adults***	486	194	---	---
***Geriatric Adults***	535	274	---	---

*** *p* < 0.05.

**Table 3:** SLP consults from each gender category.

	Gender		Total
	Male	Female	
	Number	Number	Number
**without SLP consult**	523 (51.22%)	498 (48.78%)	1021 (100%)
**with SLP consult**	275 (58.76%)	193 (41.24%)	468 (100%)

**Table 4:** Categories and gender.

Pneumonia Type	Without SLP Consult	With SLP Consult	Phi Coefficient	
			r_ϕ_	Significance
**Organism Not Specified**	651	262	0.013	0.695
***Male***	346	143	---	---
***Female***	305	119	---	---
**Health Care-Acquired**	77	32	0.110	0.250
***Male***	34	18	---	---
***Female***	43	14	---	---
**Aspiration**	88	134	0.154	0.022 ***
***Male***	47	92	---	---
***Female***	41	42	---	---
**Community-Acquired**	205	40	0.060	0.344
***Male***	96	22	---	---
***Female***	109	18	---	---
**Total**	1021	468	0.070	0.007 ***
***Male***	523	275	---	---
***Female***	498	193	---	---

*** *p* < 0.05.

**Table 5:** Logistic regression output.

Predictor	B	Wald *x*^2^	*p*	Odds Ratio	95% C.I. for Odds Ratio
**Age (1)**	−0.263	5.392	0.020	0.769	0.616–0.960
**Gender (1)**	0.317	7.819	0.005	1.372	1.099–1.713
**Constant**	−0.837	75.060	0.000	0.433	----
